# Biological and Clinical Implications of Clonal Heterogeneity and Clonal Evolution in Multiple Myeloma

**DOI:** 10.2174/157339471002141124121404

**Published:** 2014

**Authors:** Giada Bianchi, Irene M. Ghobrial

**Affiliations:** Dana-Farber Cancer Institute, Department of Medical Oncology, Harvard Medical School, 450 Brookline Avenue, Boston, MA 02215, USA

**Keywords:** Clonal evolution, clonal heterogeneity, MGUS, microenvironment, multiple myeloma, plasma cell dyscrasia

## Abstract

Clonal heterogeneity and clonal evolution have emerged as critical concepts in the field of oncology over the past four decades, largely thanks to the implementation of novel technologies such as comparative genomic hybridization, whole genome/exome sequencing and epigenetic analysis. Along with the identification of cancer stem cells in the majority of neoplasia, the recognition of intertumor and intratumor variability has provided a novel perspective to understand the mechanisms behind tumor evolution and its implication in terms of treatment failure and cancer relapse or recurrence. First hypothesized over two decades ago, clonal heterogeneity and clonal evolution have been confirmed in multiple myeloma (MM), an incurable cancer of plasma cells, almost universally preceded by a pre-malignant conditioned named monoclonal gammopathy of undetermined significance (MGUS). The genetic events and molecular mechanisms underlying such evolution have been difficult to dissect. Moreover, while a role for the bone marrow microenvironment in supporting MM cell survival, proliferation and drug-resistance has been well established, whether it is directly involved in driving evolution from MGUS to MM is at present unclear. We present in this review a historical excursus on the concepts of clonal heterogeneity and clonal evolution in MM with a special emphasis on their role in the progression from MGUS to MM; the contribution of the microenvironment; and the clinical implications in terms of resistance to treatment and disease relapse/recurrence.

## INTRODUCTION

The dynamic nature of cancer as a multistep process starting from normal tissue, *via* hyperplasia, metaplasia; localized, and eventually metastatic neoplasia, was already recognized in the late 50s [1]. This model envisioned cancer evolution mostly as a linear continuum. The first, structured theorization of cancer evolution according to the Darwinian principles of random genetic variation and natural selection in the context of limited resources, appeared on Science Journal in 1979 [2]. Dr. Peter Nowell gave voice to the exciting hypothesis that cancer originates from one founder cell, which progressively accumulates random somatic genetic mutations, thus giving rise to a series of subclonal populations existing in *equilibrium*. Such subclones compete with each other for the limited microenvironmental resources and are selected according to their fitness to survive. Similarly to what Darwin proposed in the mid 19^th^ century, cancer evolution was herein theorized as a branching rather than a linear evolution process with different subclones alternatively prevailing on the others depending on the changes in ecosystem, including exogenous perturbation such as chemotherapy [3].

Differently from the majority of hematologic neoplasia, characterized by a limited number of genetic alterations, standard cytogenetic techniques revealed early on that multiple myeloma (MM) cells presented with a significant number of karyotypic aberrations both quantitative (aneuploidy with both monosomies and trisomies noted) and structural (such as deletions and translocations), suggesting that genomic instability plays a major role in MM pathogenesis [4]. However, no distinct, recurrent, pathogenic mutation responsible for the progression from monoclonal gammopathy of undetermined significance (MGUS) to MM could be readily identified [5]. In the 1990s, analysis of DNA ploidy performed on a cell line obtained from the peripheral blood of a MM patient in the leukemic phase of the disease, clearly established for the first time the co-existence of two distinct, genetically-related clones of MM cells within the same patient and suggested that increased chromosomal abnormalities and genomic instability correlates with terminal stages of the disease [6]. These preliminary data suggested that clonal heterogeneity and evolution were present in MM.

Over the past 40 years this concept has been validated *via* genomic, epigenomic and molecular biology analysis [7]. High-resolution genetic and epigenetic analysis has been able to map the clonal evolution of a large series of neoplasms in fine details, including MM [8]. The clonal evolution theory has been refined to include the concepts of cancer stem cell and intermediate subclones with cancer stemness properties; the importance of genomic instability; the role of epigenetics; and the impact of cancer microenvironment on clonal selection [9-11].

The advent of array comparative genomic hybridization (aCGH) for copy number alteration (CNA) analysis and whole exome and/or genome sequencing (WES/WGS) provided the opportunity to further corroborate the hypothesis of clonal evolution in myeloma and deepen our understanding of the mechanisms underlying the evolution from MGUS to MM and the clinical implications of clonal heterogeneity in treatment-decision making [12]. Moreover, the relative ease of obtaining sequential samples of primary MM cells from patients evolving through different stages of the disease gave the opportunity to closely follow the natural history of alternating clonal dominance *in vivo* and correlate it with treatment response and outcomes [13, 14].

In this review, we present an excursus of the history of clonal evolution and heterogeneity in MM and focus on the contribution of the microenvironment in the process of clonal selection and competition and the biological/clinical implications of these concepts.

## FIRST CLUES TO CLONAL HETEROGENEITY AND EVOLUTION IN MM

The genomic complexity of MM cells appeared evident since the introduction of routine chromosomal analysis [15]. Complex chromosomal aberrancies have been typically associated with carcinomas, rather than hematologic neoplasia, and deemed related to early, loss of function mutations in genes controlling DNA replication fidelity and DNA repair mechanisms [16]. These observations suggested that genomic instability plays an important role in MM pathogenesis [17].

In 1979, Leibson and colleagues provided an initial clue regarding clonal evolution in MM [18]. By maintaining in culture a clone freshly isolated from the murine S107 myeloma cell line, they noted the emergence of subclonal populations over time, characterized by decreased expression of surface immunoglobulin (sIg). The authors were able to demonstrate that the difference in sIg expression was an inheritable trait, suggesting a genetic mechanism at the base of this evolution.

However, the first scientific evidence of heterogenous tumor composition in MM dates 1993 when the Mayo Clinic group performed analysis of DNA content in a cell line recently established from the peripheral blood of a MM patient in leukemic phase [6]. After establishing clonality of the cell line both at the immunoglobulin heavy chain (IgH) and light chain (IgL) locus, the authors noted the presence of two peaks of DNA content roughly corresponding to 2N and 4N when cells were analyzed *via* flow cytometry. Chromosomal analysis of metaphase cells confirmed the presence of both near-diploid and near-tetraploid karyotypes and showed that the two cell populations shared several structural and quantitative chromosomal aberrations, suggesting clonal evolution. Proliferation assay proved the near 4N population to have a faster replicative potential compared to the near 2N one. A posteriori analysis of stored bone marrow (BM) samples obtained from the patient at different stages throughout disease evolution proved the presence of both a near-2N and a near-4N clone since the first disease relapse. Of note, with each relapse, the proportion of near-4N clone tended to predominate over the near-2N, consistent with the growth advantage noticed *in vitro* and reflective of the progressively increasing biologic aggressiveness of the tumor.

It is worth noticing that this patient’s treatment included melphalan, carmustine, vincristine, doxorubicin, steroids and interferon-2α, combined in different regimens, throughout the disease course. It is plausible that in this particular patient, some of the therapy received, particularly the alkylating agents melphalan and carmustine, might have expedite the process of leukemic transformation by increasing the genomic instability of surviving MM cells. More recently, a large meta-analysis of newly diagnosed MM patients showed that lenalidomide caused an increased risk of second primary malignancies, paticularly hematologic, especially when combined with oral melphalan, suggesting that some of MM therapies can have a significant mutagenic effect [19].

## MOUSE MODEL-DERIVED DATA ON CLONAL EVOLUTION

The transgenic mouse model Vk*MYC proved helpful in following *in vivo* the natural interaction of diverse MM clones [20]. These mice sporadically develop a disease resembling human MM due to Activation-Induced Deaminase (AID)-dependent activation of the transgene MYC, expressed under the control of kappa light chain regulatory regions, during somatic hypermutation in germinal center (GC) B cells [21]. Occasionally, more than one clone of mutated GC B cell is formed, giving rise to biclonal or triclonal gammopathies. By performing BM transplant experiment between transgenic Vk*MYC mice harboring clones of different biologic aggressiveness, or between Vk*MYC mice with biclonal gammopathies and congenic C57BL/6 wild type mice, the authors showed a variable, behavior of dominant clones. Aggressive MM clones could co-exist with minor clones in *equilibrium*, promote minor subclone proliferation, or suppress it. Perturbation of this balance with anti-MM drugs modified such behavior, causing one clone to clearly prevail on the other(s) under selective pressure. Moreover, the authors observed that, depending on the genomic characteristic of each clone, either a dominant or a minor clone would survive and eventually proliferate to cause disease recurrence.

## DEEP SEQUENCING AS A TOOL TO INVESTIGATE HETEROGENEITY IN MM

WES/WGS allow examination of the coding or entire genome, respectively, and the identification of cancer-specific mutations (somatic mutations), not otherwise present in the germline of the patients harboring the disease. Compared to first generation, Sanger sequencing, second and third generation sequencing techniques allow rapid, relatively inexpensive and sensitive sequencing of modest amount of starting DNA, making them a flexible and clinically-applicable tool [22, 23]. The depth of massive parallel sequencing can be modulated in order to have either a broad, unbiased screening of the entire genome for quantitative or structural gene mutations, or a deep focus on particular areas of the genome, allowing for semi-quantitative detection of gene mutations only present in a subclonal population. It is thus evident the multitude of potential clinical applications of such techniques [24].

The group of Todd Golub was the first to perform WGS/WES of a heterogeneous group of 38 MM patients with either newly diagnosed or previously treated MM, assessed at one given point throughout the course of their illness [25]. Patients included in the study were of different age and ethnical background and some of them affected by cytogenetically high-risk [(non-hyperdiploid or harboring one of the following translocations: t(4;14); t(14;16); t(14;20) and del(17p)] MM. The results of this analysis revealed 10 genes affected by a statistically significant rate of non-silent somatic mutations. Among these, four genes had been previously identified as mutated in MM *via* standard cytogenetics: K-RAS and N-RAS (50% of samples), TP53 (8%) and CCND1 (5%).

Of the other six genes identified, DIS3 and FAM46C are of particular interest in MM as they are (DIS3), or appear to be (FAM46C), involved in the process of mRNA homeostasis and protein translation, respectively. Given the brisk synthetic activity of MM cells, ontologically derived from immunoglobulin (Ig)-producing plasma cells, both these processes are likely to be crucial in MM. Mutations affecting functionally related genes involved in the nuclear factor k B (NF-κB) and histone-modification pathways were also frequent. Moreover, activating mutations in the BRAF gene were identified in 4% of patients, thus uncovering a novel, molecular target in MM patients [26, 27].

High resolution WES of 67 patients with MM, including 15 patients for whom serial samples were available, provided interesting data regarding subclonal heterogeneity and pattern of evolution of the disease [28]. The majority of patients studied presented with advanced disease with a preponder- ance of hyperdiploid and del (17) abnormalities compared to t(11;14) or t(4;14). The data obtained with sequencing were coupled with aCGH and cytogenetics to help understand the pathogenic process in MM. First, the study showed that all tumor cells derived by each individual patient shared a set of common single nucleotide variants (SNVs), often representing the bulk of mutations, with an additional one (or more) subclone-specific cluster of SNVs. This observation confirmed the existence of a common progenitor, founder clone and the concept of clonal evolution. When specifically looking at mutations in KRAS, NRAS, BRAF, FAM46C and TP53, the authors noted that half of non silent SNVs in these genes were present in the founder clone and the remaining either appeared de novo in later disease stages, or were present in a non dominant clone that was progressively selected for, consistent with a survival advantage upon treatment. Finally, the study confirmed the presence of gain-of-function mutations in the previously known oncogenes KRAS, NRAS and BRAF in 55% of patients analyzed. Interestingly, concomitant driver mutations in more than one of these genes coexisted within the founder clone or the same subclone, suggesting that mutations in the RAS pathway are not necessarily mutually exclusive, as previously reported. Activating mutations in the MAPK pathway were also reported and again more than one signaling molecule was found mutated within the same subclone. Mutations in FAM46C, in a pattern suggestive of onco-suppressor function, were detected in 12% of patients and associated with hyperdiploid karyotype (p value, 0.02). Finally, several, novel candidate genes were identified during this study including SP140, a homolog of SP100 with restricted expression in lymphoid cells, whose function is not completely understood but appears related to antigen response in B cells; and LTB, a transmembrane protein of the TNF superfamily, involved in lymphoid differentiation through NF-κB signaling pathway. In light of their putative function, pattern of somatic mutations (largely truncating), and association with loss of heterozygosity of LTB in two third of the patients, these two genes appear putative onco-suppressors in MM. Several MM patients also harbored mutations in: ROBO1, a transmembrane receptor central in migration and neuronal development, previously reported mutated in pancreatic adenocarcinoma; FAT3, a transmembrane protein of the cadherin superfamily of endothelial junction molecules; and EGR1, a transcription factor controlling mitosis and cell differentiation. These molecules thus represent potential novel targets in MM. Of note, the presence of mutations in any of these genes did not have any impact on overall survival (OS) while mutations in TP53 and SP140 correlated with worse progression free survival (PFS), suggestive of a potential prognostic role.

Preliminary data on single cell genetic analysis of primary MM cells confirmed the presence of double-hit mutations in KRAS or KRAS/NRAS as well as concomitant mutations in RAS and MAPK pathway [29]. Specifically, the authors reported parallel evolution of distinct subclones derived by the same progenitor, which independently acquired mutations in the RAS pathway. These data are consistent with mutations in RAS being driver rather than passenger mutations and suggest a survival advantage of clones harboring such genomic abnormalities.

The results of these studies confirmed the previously suspected inter-tumor heterogeneity of MM and paved the way to outline the presence of clonal evolution by longitudinally sequencing MM cells obtained from the same patient at different time points during the course of the disease.

## LONGITUDINAL FOLLOW UP OF PRIMARY MM CELLS REVEALED DIFFERENT PATTERNS OF CLONAL EVOLUTION IN MM

The Mayo clinic group first reported on aCGH performed on serially obtained paired samples of 28 patients with either high or standard-risk cytogenetic MM [20]. Analysis of CNAs revealed three distinct patterns of evolution: 35.7% of patients showed stable CNAs throughout a median follow up of 13.3 months; 21.4% only acquired new CNAs and the remaining 42.9% showed both gain and loss of CNAs over time. In this latter group, regions of the genome that were homozygously deleted in the initially dominant tumor clone, reappeared over the course of the disease, consistent with the theory of clonal heterogeneity with alternate dominance. Of note, these three patterns were not equally represented in high-risk and low-risk patients: rather, tumor cells characterized by high-risk cytegenetics showed a significantly higher frequency of changes in CNAs over time, reflecting increased clonal heterogeneity, likely related to genomic instability. Moreover, patients with del(17p) displayed a significantly higher CNAs at the time of diagnosis compared to other high risk patients, consistent with TP53 being a major gatekeeper of DNA stability.

Similarly, tumor and germline DNA obtained from a 67 year old woman newly diagnosed with t(4;14) and del(13) MM was analyzed longitudinally *via* WGS. Matched samples were obtained at time of diagnosis, first and second relapse and eventually progression to secondary plasma cell leukemia, over a period of four and half years [14]. The authors identified 124 somatic, non-synonymous, SNVs affecting exons, 36 of which were validated with Sanger sequencing. Twenty-seven out of these 36 genes were already reported as mutated in the previously mentioned study or in the COSMIC database, consistent with a putative role in oncogenesis [30]. Interestingly, 10 SNVs were carried along in the tumor specimen, throughout the disease evolution, suggesting a role as driver mutations, while others were detected only in two out of four samples, consistent with the presence of subclones with alternating dominance during the course of the disease. In analogy to previous reports by other groups, the sample obtained in the terminal phase of the disease, at the time of secondary plasma cell leukemia evolution, was characterized by a significantly higher amount of genetic aberrancies, and distinct, unique SNVs, not previously noted in the tumor samples. These observations suggest that genomic instability is a central process in the terminal phase of MM and that certain discrete mutations might be responsible for the leukemic transformation of the disease.

**Fig. (1):**
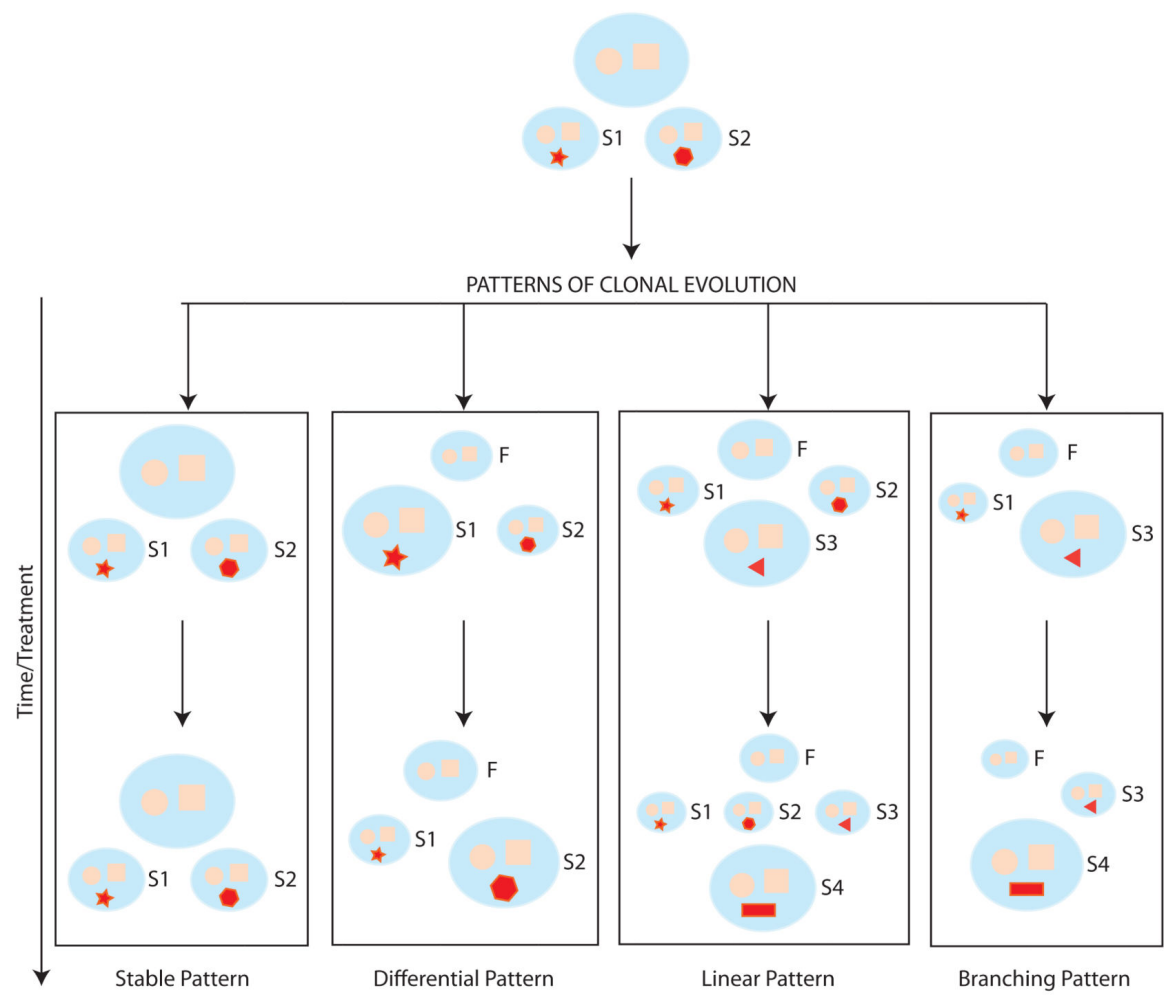
Patterns of clonal evolution in MM The cartoon is a schematic representation of the diverse patterns of clonal evolution as described by Bolli *et al.* MMCs are represented in pale orange. The blue circle and square represent the mutations of the founding (F) clone, while the various red, geometric shapes reflects acquired, somatic mutations within a subclone (S1, S2, S3 and S4). The size of the clone is representative of the relative dominance within the tumor population, with the largest clone representing the dominant one. Each pattern is represented within a rectangle with the Y axis representing time. Abbreviations: F: founder clone; S: subclone

In their study, Bolli and coauthors also reported on the process of clonal evolution by deep sequencing of the 15 patients for whom serial samples were available [28]. Four different pattern of evolution were identified (Fig. **1**). A third of the patients showed no changes in the pattern of mutations and the relative abundance of subclonal populations over time, despite patients undergoing treatment. This pattern of evolution (or rather, non-evolution) was more common among patients with t(11;14) than individuals harboring a hyperdiploid karyotype (80% versus 11%, respectively, p value 0.023). In 27% of patients, different subclones demonstrated alternative dominance over time and post treatment, suggesting either diverse sensitivity to therapy, selective advantage of one clone versus the other, or, less likely, a random change of dominance pattern; 13% of patients demonstrated a linear evolution pattern with a new subclone, not previously detected, becoming dominant post treatment; the reminder of patients had a branching pattern of clonal evolution with new clones emerging and other declining or disappearing over time. This pattern was particularly common in patients relapsing with extramedullary disease whose dominant clone was typically characterized by profound genomic changes compared to the founder clone. De novo mutations in the RAS and NF-κB pathways and loss of function mutation in TP53 and FAM46C were commonly found at this stage. We will discuss the impact of therapy in this selection process in a later section.

## CLONAL HETEROGENEITY AS A TOOL TO UNDERSTAND MM PATHOGENESIS AND PROGRESSION FROM MGUS TO MM

It was recently reported that MGUS, a precancerous condition characterized by 1%/year rate of progression to neoplasia, consistently precedes MM [31, 32]. The molecular mechanisms underlying the progression of the disease are largely obscure and genomic studies to date have been unable to identify consistent, unique, driving mutations. Indeed, translocation between the IgH enhancer locus and recurrent partners (such as FGFR3/MMSET; CCND1 and MAF), which are characteristic of non-hyperdiploid MM patients, were reported to be already present in MGUS patients [33, 34]. However, the frequency of such translocations as well as other cytogenetic abnormalities, such as del [17], del [13], (mutated chromosomes) gain 1q and del(1p) occurred with significant lower frequency in MGUS compared to smoldering MM (SMM) and MM patients and proportionally affected less tumor cells compared to more advanced dyscrasia [35, 36]. Moreover, clonal heterogeneity was recently shown to be an early event in plasma cell dyscrasia and present both in MGUS and SMM patients, although the number of non-synonymous SNVs appears to increase progressively as MGUS evolves into MM and eventually plasma cell leukemia [37].

Analysis of peripheral blood from over 450 patients with plasma cell dyscrasia in different disease stage revealed the presence of a variable proportion (0.01%-61% of total leukocyte count) of circulating MM cells. The proportion of patients with circulating cells was higher in patients with newly diagnosed and/or relapsed MM (63.4% and 64.5%, respectively) as compared with patients with MGUS and/or SMM (25% and 24%, respectively). WGS of paired peripheral blood, BM and germline samples showed that marrow-derived and circulating MM cells only shared 5-38% mutations, with evidence of acquisition of driving mutations such as BRAF V600E, in the circulating tumor cells. These data suggest that the process of re-circulation outside the BM niche entails clonal evolution with acquisition of a substantial amount of novel genetic mutations, likely crucial for the process of MM trafficking and progression [38].

As plasma cells are the product of terminal differentiation of B lymphocytes, MM-initiating mutations could potentially occur anywhere along the lymphopoieitic process [39]. The events occurring at the germinal center, in particular isotypic class switch and somatic hypermutation, raised interest as physiologic processes of DNA re-arrangement during which potentially oncogenic events could occur [40]. Analysis of the variable heavy chain (V_H_) sequence in MM was consistent with clonal, post GC origin, with no evidence of ongoing hypersomatic mutation in the V_H_ region [41]. Vice-versa, a certain proportion of MGUS cells showed ongoing, intraclonal variability of the V_H_ region suggesting persistent somatic hypermutation and possibly continuous recirculation between the GC and the BM [41, 42]. Progression to MM appears to abate the ongoing somatic hypermutation process noted in MGUS and entails the selective advantage of one single clone, likely secondary to acquisition of further somatic mutations, which becomes dominant and characterized by a unique, consistent V_Hsequnce_. However, most recently, deep sequencing of the Ig locus in 193 primary MM cells revealed presence of oligoclonality in 12% of patients. In two thirds of cases, the Ig sequence was related, consistent with evolution from the same founder clone. Isotype switch and somatic hypermutation were deemed to be at the base of oligoclonality in 73% and 27% of patients analyzed, respectively, suggesting that these process can be re-activated after evolution from MGUS to MM [43]. Although these data clearly outline a difference between MGUS and MM cells, they do not clarify whether the driver mutation for MM evolution is acquired in the GC or in the BM niche.

## INVESTIGATING DIFFERENCES IN CLONAL EVOLUTION IN PATIENTS WITH HIGH-RISK VERSUS STANDARD-RISK CYTOGENETICS

Walker *et al.* utilized the WES technology to address whether differences existed in terms of clonal evolution in patients with high-risk [t(4;14)] versus standard-risk [t(11;14)] cytogenetics [44]. Former patients showed a trend toward higher SNVs compared to latter patients, although not reaching statistical significance. While both groups shared some common, highly mutated genes such as KRAS and DIS3, only 3% of the overall mutated genes in each group overlapped. Such shared genes largely encoded for proteins involved in cell-cycle regulation, proliferation and plasma cell differentiation. By performing gene ontology analysis for each group, samples from patients harboring t(4;14) were enriched in genes involved in microtubulin-based transportation, actin-based movement and cytoskeleton organization as well as chromatin remodeling, consistent with a major role for epigenetic mechanisms. Patients with t(11;14) showed a prevalence of genes involved in phosphorylation and phosphate metabolism as well as Rasmediated signaling, including 4% prevalence of BRAF mutations. Of note, while activating mutations in the RAS-MAPK signaling pathway have been well established as central for oncogenesis, deep genome analysis clearly established the presence of subclones not harboring such mutations which were dominant in about half of patients. Moreover, mutations along the same axis were noted to be mutually exclusive, suggesting no survival advantage for clones harboring mutations in more than one molecule along this signaling pathway. However, co-exisitng mutations were found in a different study [28]. Overall, this study confirms the concept of clonal heterogeneity in MM and establish different patterns of gene mutations for MM patients with t(4;14) and t(11;14), suggesting that genes differentially expressed by the former patients might be responsible for the aggressive biology of their cancer and their dismal outcome.

## THE IMPACT OF THE BONE MARROW MICROENVIRONMENT ON CLONAL EVOLUTION

Growing evidence supports a pivotal role of the microenvironment in tumorigenesis and tumor progression [45]. Cancer niches have been clearly shown to promote tumor proliferation, metastasis, resistance to therapy and eventually recurrence/relapse in a number of cancers, including MM [46]. It has become evident in recent years that cancer cells and mesenchymal/stromal cells, not only spatially interact *via* adhesion molecules with subsequent bidirectional signaling, typically resulting in a survival advantage for cancer cells, but also exchange macromolecules such as nucleic acid and proteins *via* microvesicles and exosomes [47-49]. Roccaro and colleagues recently showed that exosomes released by bone marrow mesenchymal/stromal cells can be up-taken by MM cells and results in their increased proliferation [50]. The authors proved that microRNAs (miRs) could be successfully transferred from exosomes to MM cells, resulting in epigenetic modulation of target genes, and that the miR and protein content of exosomes differed significantly between patient- and healthy control-derived BM mesenchymal/stromal cells. In particular, miR-15a, an established onco-suppressor in MM, was found to be significantly down-regulated in exosomes released by cancer-derived BM mesenchymal/stroma cells in comparison to their normal counterpart [51]. MM cells co-cultured with cancer-derived BM mesenchymal/stroma cells showed a decreased level of expression of miR-15a compared to cells co-cultured with normal stromal cells, suggesting that the microenvironment plays an important role in epigenetically modulating gene expression. These exciting data pave the way to explore the possibility of a role for the microenvironment in guiding clonal evolution and heterogeneity in MM. Indeed the exact nature of the material exchanged *via* exosomes between cancer cells and the tumor niche has not been completely elucidated and exchange of DNA, in particular oncogenes has been proposed in solid tumors.

Altogether, these data provide a further layer of complexity regarding the dynamic nature of cancer cell genomics and the impact of BM niche in guiding clonal evolution in MM and increased therapeutic resistance [52].

## THE CLINICAL REPERCUSSIONS OF CLONAL HETEROGENEITY IN OUTLINING MYELOMA TREATMENT

WGS of patient samples obtained at different time points over the course of MM evolution offers useful data regarding the effect of specific treatment strategies on subclones harboring diverse mutations. The previously discussed report on the longitudinal follow up of a newly diagnosed patients with t(4;14), revealed several important information [14]. The disappearance and dominance of different clones in this patient appeared to be clearly determined by selective pressure from treatment. For instance, the first relapse of the patient after a partial remission with lenalidomide and low dose dexamethasone (Rd) was attributed to the emergence of a previously minor clone, which became progressively dominant as the founder clone declined under the pressure of treatment. Both this clone and a related one that appeared at this time harbored mutations in the BIRC2/3 gene, a positive regulator of the NF-κB pathway, suggesting a role for NF-κB in mediating Rd resistance. This data suggest that partial response can be the consequence of lack of suppression of a non-dominant clone rather than to partial suppression of the whole tumor population and provide a strong biologic rationale for using combinatory chemotherapy in an attempt to eradicate all clones and avoid selection of aggressive ones. Interestingly, at the time of the fourth relapse after receiving melphalan, prednisone and bortezomib (MPV) therapy, the dominant clone was profoundly different from the founding clone and characterized by complex genomic abnormalities, raising the possibility that melphalan, an alkylating agent, could potentially select for highly genomically unstable and aggressive clones. Eventually, a triploid version of this highly unstable clone was responsible for progression to plasma cell leukemia and patient demise.

## CONCLUSIONS AND FUTURE DIRECTIONS

The understanding of genetic events at the base of tumorigenesis, cancer progression and metastasis has greatly benefitted by the introduction of sensitive and accurate sequencing techniques of the genome and epigenome. Such tools have helped confirmed the theory developed in the 1970s-1980s of branching evolution of cancer cells where random genetic mutations are selected for and perpetrated along according to a selective advantage in a ecosystem (cancer microenvironment and more broadly the human body) with limited resources.

From the first clues in the 1990s to today, clonal heterogeneity and evolution have been proven to occur also in MM, an incurable cancer of terminally differentiated plasma cells (Table **1**). Comparative analysis of WES/WGS of primary cells obtained from standard-risk and high-risk patients as well as sequential analysis of patient’s derived MM cells throughout the course of the disease have been instrumental in further understanding the biology of MM and potential genetic mechanisms at the base of cancer progression. Moreover, deep sequencing of pre-malignant plasma cell dyscrasias such as MGUS and SMM and comparison to MM samples has shed light on the increased genomic instability and clonal heterogeneity characteristic of disease transformation. More recently, the proof of exchange of miRs between BM stromal/mesenchymal cells and MM cells clearly established a role for the BM microenvironment in epigenetically controlling MM cell fate. It is reasonable to hypothesize that exosomes could be responsible for the exchange of genomic DNA and potentially oncogenes, thus adding a layer of further complexity to the concept of clonal heterogeneity in MM.

However, despite the sensitivity of deep sequencing techniques, specific, recurrent genetic driver mutations have not been clearly identified in MM. Epigenomic analysis of primary samples from patients with chronic lymphocytic leukemia (CLL) has provided important information regarding the differences between normal and malignant lymphocytes, showing widespread DNA hypomethylation prevailing in the latter [53]. Moreover, different biological and clinical categories of CLL reflected in a diverse epigenomic signature profile, suggesting epigenomic analysis as a valuable tool to help design personalized treatment. Preliminary data on DNA-damage and repair pathways and epigenetic changes in MM helped not only shedding light on the biology of MM, but also identifying potential druggable targets for MM treatment [54, 55]. Further information regarding epigenomic in MM and precursor dyscrasia is eagerly awaited in the hope that it could help our understanding of MM etiopathogenesis and the design of more effective treatment strategies.

## Figures and Tables

**Table 1:** Landmark publications regarding the concept of clonal heterogeneity and evolution in MM. The table summarizes the most relevant studies in the field of clonal heterogeneity/evolution in MM

Authors	Year	Study Design	Findings
Leibson *et al*.	1979	Serial flow cytometry analysis of sIg expression of a murine myeloma cell line cultured over time	Emergence of a subclone of cells characterized by inheritable, lower expression of sIg
Jelinek *et al*.	1993	Ex vivo analysis of ploidy in stored BM aspirate samples and a clonal cell line established from a MM patient with secondary PC leukemia	Co-existance of 2N and 4N, distinct, but genetically-related clones Dominant clone in leukemic phase showed increased genomic complexity and proliferative advantage
Sahota *et al*.	1996	V_H_ sequence analysis in MGUS and MM patients	A proportion of MGUS cells showed ongoing hypersomatic mutation MM clones showed no ongoing hypersomatic mutation
Chapman *et al*.	2011	WES/WGS of 38 MM patients	Increased rate of somatic mutations identified in K-RAS, N-RAS, TP53, CCND1, DIS3, FAM46C BRAF mutations in 4% patients High rate of mutations in genes involved in NF-κB signaling and histone modification
Keats *et al*.	2012	Bone marrow transplantation between Vk*Myc mice harboring mono- clonal/biclonal gammopathy and or between Vk*Myc and congenic C57BL/6 mice	Clonal dominance is determined by genetic asset of the clone and the selective pressure of the cancer environment Aggressive clones could suppress or enhance proliferation of minor clones or coexist in equilibrium Exogenous perturbations such as chemotherapy alter this behavior
		aCGH in serial samples of 28 MM patients	Three pattern of evolution identified: no changes in CNAs; only gain of CNAs; both gain and loss of CNAs over time. High-risk cytogenetic patients showed increased frequency of CNAs over time compared to standard-risk ones Del(17) associated with higher CNAs at diagnosis
Egan *et al*.	2012	WGS of serial samples from a patient witht(4;14) and del(13) MM from diagnosis till progression to secondary PC leukemia	10 SNVs were present from diagnosis till leukemic phase Certain SNVs appeared and disappeared in serial samples Chemotherapy major determinant of clonal dominance *via* selective pressure Dominant clone post melphalan therapy characterized by complex genomic abnormalities Leukemic phase clone showed increased genomic instability
Walker *et al*.	2012	WES in MM patients with t(4;14) versus t(11;14)	Only 3% of mutated genes overlapped between the two groups T(4;14) associated with mutations in genes involved in microtubule transport, actin and cytoskeleton organization and chromatin remodeling T(11;14) associates with genes involved in phosphorylation and phosphate metabolism and Ras pathway BRAF mutations associate with t(11;14)
Roccaro *et al*.	2013	Analysis of content of BMSC exosomes derived from MM and healthy donor and their effect on MM cell proliferation and neoplastic poten- tial	MM cells are capable to uptake exosomes relased by BMSC Uptake of MM patient-derived BMSC exosomes causes increased proliferation of MM cells The content of BMSC-derived exosomes differs between healthy donor and MM patients miR-15a, an oncosuprressor, is downregulated in BMSC exosomes from MM patients
Walker *et al*.	2014	WES/WGS of patients with plasma cell dyscrasia: MGUS, high-risk SMM, MM and PC leukemia	Clonal heterogeneity already present in MGUS and SMM Number of non-synonymous SNVs increased during PC dyscrasia progression
Bolli *et al*.	2014	WES of 67 MM patients, including 15 patients with longitudinal follow up	Common set of SNVs present in all MM cells of a single patient Mutations in K-RAS, N-RAS, BRAF, TP53, FAM46C present in 50% founder clones Gain of function mutations in K-RAS, N-RAS, BRAF occurred frequently (55% of patients) and could coexist within same clone Mutations in MAPK pathway occurred frequently and coexisted within same clone Mutations in FAM46C consistent with oncosuppressor function and associated with hyperdiploid karyotype SP140, LTB, ROBO1, FAT3 ad EGR1 identified as novel gene candidate Four different patterns of clonal evolution identified (Fig. 1)

Abbreviations: sIg: surface immunoglobulin; PC: plasma cell; BM: bone marrow; MM: multiple myeloma; WES: whole exome sequencing; WGS: whole genome sequencing; MGUS: monoclonal gammopathy of undetermined significance; aCGH: array comparative genomic hybridization; CNA: copy number alteration; SNV: single nucleotide variant; BMSC: bone marrow stroma cells; miR: microRNA

## LIST OF ABBREVIATIONS

MM: Multiple myeloma
MGUS: Monoclonal gammopathy of undetermined significance
aCGH: Array comparative genomic hybridization
CNA: Copy number alteration
WES: Whole exome sequencing
WGS: Whole genome sequencing
Ig: Immunoglobulin
sIg: Surface immunoglobulin
IgH: Immunoglobulin heavy chain
GC: Germinal center
AID: Activation-Induced Deaminase
MYC: v-myc avian myelocytomatosis viral oncogene homolog
K-RAS: Kirsten rat sarcoma viral oncogene homolog
N-RAS: Neuroblastoma RAS Viral (V-Ras) Oncogene Homolog
TP53: Tumor protein 53
CCND1: Cyclin D1
DIS3: DIS3 exosome endoribonuclease and 3′-5′ exoribonuclease
FAM46C: Family with sequence similarity 46, member C
NF-κB: Nuclear factor κ B
BRAF: v-raf murine sarcoma viral oncogene homolog B
SNV: Single nucleotide variant
MAPK: Mitogen-activated protein kinase
SP140: SP140 nuclear body protein
SP100: SP100 nuclear antigen
LTB: Lymphotoxin beta (TNF superfamily, member 3)
TNF: Tumor necrosis factor
ROBO1: Roundabout, axon guidance receptor, homolog 1
FAT3: FAT atypical cadherin 3
EGR1: Early growth response 1
OS: Overall survival
PFS: Progression free survival
FGFR3/MMSET: Fibroblast growth factor receptor 3/multiple myeloma SET domain containing protein
V_H_: Variable heavy chain region
BIRC2/3: Baculoviral IAP repeat containing 2/3
miR: microRNA
CLL: Chronic lymphocytic leukemia
